# Of the Osseous Union of Teeth

**Published:** 1839-12

**Authors:** Solyman Brown


### OF THE OSSEOUS UNION OF TEETH,

[ BY SOLYMAN BROWN. ]

As Mr. Parmly's specimen of this phenomenon, described in a former number, is of the most decisive and convincing character, precluding the possibility of controversy or doubt on the subject in the mind of any person who inspects it; it may be well to bring together two or three authorities on this occasion, which I think will set the question permanently at rest. At any rate, something more demonstrative than mere scepticism on the subject will be hereafter required to justify any writer in asserting, that "there is no other way of accounting for such a doctrine, than by attributing it to a weak credulity, or love of the marvellous, or a desire to impose upon the world."

The following remarks are from the valuable work of Mr. Thomas Bell, lecturer on the anatomy and diseases of the teeth, at Guy's Hospital, London, page 106, of the Philadelphia edition by Carey & Lea, 1830.

"The intimate and inseparable connexion of two teeth, by means of a true bony union of their roots and sides, though not a frequent occurrence, is too well established by facts, to admit of a moment's doubt. I have met with four instances of it in my own practice, in three of which the temporary superior central incisores were the subjects of this connexion. There are also several interesting specimens of it in the collection at

Guy's Hospital, some of which are figured in Mr. Fox's work. There is no reason to suppose that this union ever takes place after the formation of the teeth has been completed, by the deposition of new bone upon the parts in apposition: in some cases, as in the central incisores, it may doubtless be produced by the original union of the two pulps, previous to the production of bone upon their surface; but, in others, where those teeth are found united, the pulps of which were not strictly contemporary with each other—as in the union of a bicuspis with a molaris—the connexion must have been formed during the progress of their ossification.

This deviation from the natural condition of the teeth cannot, unfortunately, be known by any peculiarity in their form or position whilst they exist in the jaw; and it is only by the lamentable result of an attempt to extract a tooth so circumstanced, that it can be ascertained; for it is obvious that such an operation must be attended, either by the removal of both, or by the fracture of one of them. The case, however, is of extremely rare occurrence, and we must attribute most of the instances which are related, either to a want of discrimination on the one hand, or, on the other, to an attempt to excuse an ill-performed operation.*

---

* As this bony connexion of two teeth is a purely accidental occurrence, and unconnected with any other circumstance of a practical nature, than that which I have mentioned, I should not have thought it necessary to enter more into the detail of the cases I have enumerated above, or to endeavor farther to establish their truth than by my own assertion of their having occurred, were it not for an extraordinary attempt which has lately been made, to deny the existence of such cases, and to throw more than doubt upon the veracity of Mr. Fox's statements. I allude to the following passage.

"For my own part I must declare, that during *all* my practice for many years, I have never been able to obtain ocular demonstration of such a fact, or to satisfy myself that there ever has been such a case. And this I say, also, of all my professional brethren with whom I have had an opportunity to converse on the subject. I hope, therefore, that my scepticism on this point will not be construed into a want of becoming respect for the authorities from which these cases are derived. There is no other way of accounting for such doctrine, than by attributing it to a weak credulity, or love of the marvellous, *cr a desire to impose upon the world.* Let us take into consideration one of these cases. Mr. Joseph Fox mentions an instance of this supposed union in two central incisores of the upper jaw at their contiguous sides."

Then follow some observations on the probabilities of the case, which do not bear upon the point I am now discussing. The author then proceeds:—

"I indeed believe that Mr. Fox *did not himself see the case he thus describes*, else he would assuredly have given a more circumstantial account of it. Such cases furnish a fine apology to ignorant operators, and I have no doubt that the first case of this kind originated in some bungling accident or ingenious deception," &c.—Koecker's Dental Surgery, p. 319.

The character of Mr. Fox happily stands too high in professional estimation, to require any laboured defence on my part; and an attempt to impugn the veracity of a man whose character was almost proverbial for simplicity and truth, can but recoil with redoubled force upon his accuser. I waive the circumstance that this attack is made on one who is no longer living to rebut the charge, for it fortunately happens that some of the identical specimens from which Mr. Fox's descriptions and figures were taken, are, at the present moment, in the museum at Guy's Hospital, and have been constantly exhibited in my lectures on the teeth, in the theatre of that school, for the last ten years. Mr. Fox has figured

To these two conflicting authorities, I shall add a translation from the "SURGEON DENTIST, or Treatise on the teeth," by Pierre Fauchard, published in Paris more than a hundred years ago. Volume 1, page 342, chap. XXVII. "Four observations concerning teeth variously united together."

First observation. Of two carious teeth so united as to constitute but one body; extracted together.

"In 1705 a Franciscan Friar of the city of Lude in Anjou, came to me to have a large molar tooth extracted, which had caused him much pain. I examined his mouth; discovered that the tooth was very much decayed, and that there was no other course fitted to give him relief, but to execute his design. Although I siezed with the instrument which I used on this occasion, only the one tooth which I wished to extract, yet I nevertheless drew two at once. I thought at first that I had committed an egregious error, but I found that the tooth which had followed the first, was decayed as well as the other, and that they both adhered so closely by an union of their roots, that they formed almost one and the same body. The Friar thinking that I was deceived on the subject, had the curiosity to examine and ascertain the truth of the assertion. In order the better to convince ourselves, we took a knife, the blade of which we placed across the two teeth, and struck upon it with a stone. By this method we could not succeed in separating the two teeth, but by breaking them into fragments, which satisfied the recluse that it was impossible to extract the one without the other. The trouble which I took to instruct the Friar in a truth equally instructing to us both, caused us to part good friends.

REFLECTION.—When teeth are united by their roots only, it cannot be known until after they are extracted. It is different when they are united by their crowns. In this latter case, we ought, before we operate, to inform the persons who have such teeth, that one cannot be taken out without

---

three of these cases, though the author in question has quoted but one, and that, certainly, the only one which, from its character and appearance *as figured*, could admit of a cavil. I can, however, vouch for the correctness of this as well as of the others, and without hesitation assert that they are true, undeniable instances of the bony union of teeth.

With regard to the assurance, that "in *all* his practice for many years, the author has never been able to meet with ocular demonstration of such a fact," it really amounts to nothing; but when he declares that "all his professional brethren with whom he has had an opportunity to converse on the subject," have been similarly circumstanced in this respect, I feel myself called upon to remind that gentleman of the following facts which, in the course of years, have undoubtedly passed from his memory.

Mr. Koecker will perhaps remember that, on a temporary visit to England, he did me the honor to call on me, with an introductory letter from a gentleman of the highest eminence in the profession; and that, in consequence of that introduction, I had the pleasure of shewing him my collection of preparations, illustrative of the anatomy and diseases of the teeth, at Guy's Hospital, the major part of which had been collected by Mr. Joseph Fox; and that amongst those which then occupied our attention, were the *very specimens* to which I have just alluded. I am quite sure that had Mr. Koecker recollected this circumstance, he would have had the candour to omit the passage which I have quoted; and I trust that my motive in having endeavored to elucidate the question, will be appreciated as originating only in a sense of justice, and an anxious desire to rescue deceased worth and talent from posthumous misrepresentation."

the other. By this means we avoid all discussion; but when we cannot know the secret adhesion until after extraction, we should, as soon as we discover the union, inform the patient, to justify ourselves in his opinion, lest he should impute to our art, or to defect of experience therein, an accident which depended wholly on natural peculiarity in the case itself."

*Second Observation.*—Of two teeth united together so as to form but a single crown.

"On the 20th of December, 1723, Miss Lemoyne, aged eight years, residing in Paris near Saint *Magloère,* was brought to my rooms much distressed with tooth ache. On examining her mouth, I found that the canine and its neighboring incisor on the right side of the lower jaw, were so closely united that they formed but one body. Between these two teeth there appeared a species of gutter of little depth, along the crown, with a small interval at the extremity. This double tooth was composed of two milk teeth very strongly united. I did not extract them for fear of injuring the germ which lay beneath, and which should naturally produce their successors of the permanent set."

*Third Observation.*—Somewhat similar to the preceding.

On the 16th of January, 1724, I went to the house of Mr. Auger, a wholesale grocer. I examined the teeth of his daughter, about eight years old, and remarked, that the lateral incisor of the right side of the upper jaw, belonging to the temporary set, was united to the adjoining cuspidatus, which is not a very common occurrence. I pointed it out to the father and mother, to Mr. *Dandreau,* auditor of accounts, and to several other persons present.

Reflection.—It is difficult to determine, whether or not the union of teeth, which are found joined together, depends on the blending of the germs: The middle partition of two of the alveoli not being yet formed, these two constitute but one cavity, and consequently a double tooth, or twins, may, in such case, be the result. It is always embarrassing to have such teeth, because if one of them should be injured by any accident, the other is in imminent danger of being also destroyed

*Fourth Observation.*—Concerning a sound tooth which it had been thought necessary to extract with an adjoining carious tooth, because they were both attached to the intervening paries of the alveolus.

In 1711, a master cordwainer, requested me to extract the first small molar tooth on the right side of the upper jaw. This tooth was carious and occasioned insufferable pain. Although it appeared to me to be a very difficult tooth to extract, I did not let the patient know it—and succeeded in the operation. Fortunately, I discovered, during the operation, that the small molar (bicuspid) started from the alveolus, together with that which I wished to remove. In an instant I withdrew the instrument, judging by this that the middle portion of the alveolus was firmly attached to these teeth, and that this partition was separated from the rest of the alveolus, by the effort which I had been compelled to make. As soon as I discovered this, I re-placed both of the teeth in their cavities, and separated with a file the parts of the alveolus which held them together. By this means I succeeded in removing the carious tooth without difficulty, and its neighbor which had been disturbed was adjusted in its original position. If I had not had recourse to this expedient, I should have made a great breach in the alveolar process as well as the gum; and moreover, I should have removed a good tooth, which could not have failed to follow the bad one.

Reflections.—It happens every day, in taking out teeth, that we meet with difficulties which cannot be foreseen. If there be any method of shunning the accidents which may possibly occur, it must be by operating prudently and without precipitation. It is necessary to be cautious in giving the first motion to a tooth, and to observe carefully the resistance which it makes to the power applied; above all, to be attentive to what passes, in regard to the other teeth.

If the operator discovers that these are shaken, he may infer that they touch, in some point, the tooth to be extracted. If the agitation is very considerable, there is a possibility that the several teeth are united together, either by intervening alveolus, or by some other means. In such a case, it is necessary to proceed as I have described; and when the operator is well informed, circumspect and ingenious, he will not only avoid many accidents, but introduce into his practice, modes of operation from which the public shall derive the greatest advantage."

Thus speaks Fauchard, of operations performed in the early part of the last century, and of cases of unquestionable osseous union; and the fact that the fourth example given by him, proved to be of another character, viz: the adhesion of two adjacent teeth to the intervening plate of the alveolar process, serves rather to corroborate than weaken the testimony of the author. If Fauchard had not thus put us in possession of the fact that he was well acquainted with cases in which apparent and not real union has place, we might possibly surmise that he had mistaken the character of the examples to which he has referred.

To conclude, I may be permitted to remark, that the scepticism of individuals, however wise or learned, in relation to natural phenomena by them unobserved, is of too negative a character to be the test of scientific truth.

